# “Three Steps Above Heaven? Really? That’s All Tactic!” New Alternative Masculinities Dismantling Dominant Traditional Masculinity’s Strategies

**DOI:** 10.3389/fpsyg.2021.673829

**Published:** 2021-06-11

**Authors:** Roseli Rodrigues-Mello, Lars Bonell-García, Marcos Castro-Sandúa, Esther Oliver-Pérez

**Affiliations:** ^1^Department of Pedagogical Theories and Practices, Centre of Research and Social and Educational Action, Federal University of São Carlos, São Carlos, Brazil; ^2^Department of Social Work and Social Education, Centro Universitario La Salle, Madrid, Spain; ^3^Department of Education, Nebrija University, Madrid, Spain; ^4^Independent Researcher, University of Barcelona, Barcelona, Spain; ^5^Department of Sociology, University of Barcelona, Barcelona, Spain

**Keywords:** communicative acts, dominant traditional masculinities, new alternative masculinities, language of desire, violence, preventive socialization

## Abstract

Research on preventive socialization of gender violence has contributed abundant empirical evidence that attraction to violence is common among adolescents. This has meant that “bad guys,” or those who reflect the Dominant Traditional Masculinity (DTM) model, are chiefly perceived as appealing, while “good guys” are perceived as good friends but not desirable. The mainstream media tends to reproduce this traditional model of affective-sexual relationships, which has harmful effects on young girls concerning gender and sexuality. However, New Alternative Masculinity men are challenging this traditional and unsatisfactory model of affective-sexual relationships. The 2010 Spanish version of the movie Three Steps above Heaven, a good example of this kind of media product, has proven to greatly impact communicative acts among adolescents. This article explores how this influence on adolescents is because the communicative acts about Hache – the main character in the movie – are full of the language of desire, and his own communicative acts are full of violence. On the one hand, we analyze how Three Steps above Heaven employs communicative acts to enhance the attractiveness of DTM. On the other hand, based on the evidence gathered in a communicative focus group (CFG) addressed to 15- and 16-year-old female adolescents, we analyze how New Alternative Masculinity men are demystifying Hache and the idea of having a “Three Steps Above Heaven” by demonstrating with the powerful language of desire that men like him employ farce strategies. The article includes evidence from interventions with adolescents where discussion of movies like this, with the involvement of New Alternative Masculinity men and grounded in the language of desire, can transform the perception about the sexual-affective relationship in the movie, thus counteracting their negative influence in terms of attraction to violence.

## Introduction

Violence in affective-sexual relationships is a global public health problem that affects women of different ages and socio-economic contexts (United Nations Statistics Division, 2015), both in stable and sporadic relationships (Puigvert et al., 2019). Gender-based violence affects young women to a greater extent (Stöckl et al., 2014). Having suffered violent relationships entails a series of severe health risks, including symptoms of depression and anxiety and suicidal ideations (Exner-Cortens et al., 2013). One of the risk factors that increase the chances of suffering violence in affective-sexual relationships is the influence of a dominant coercive discourse that connects attraction with violent attitudes and behaviors (Puigvert et al., 2019). This discourse is disseminated through the media and the primary agents of socialization (Gómez, 2014).

For many Spanish adolescents, the 2010 Spanish version of the Italian movie Three Steps above Heaven (from now on, 3MSC, from the Spanish title 3 Metros sobre el Cielo), originally a book written by Federico Moccia, is the most, or at least, one of the most exciting love stories they have ever known. Additionally, Hache, the main male character, is one of the most appealing boys and, by extension, Mario Casas, the actor performing this role, has become an idol for teenagers. For example, one of the posts about this film in the most popular Spanish magazine for adolescents revealed how teenagers’ communicative acts considered both Hache and his affective-sexual relationship with Babi (the girl) as examples of excitement and desire. This is also present in the large amount of 3MSC-related content created by Internet users such as comments, photomontages, or even Twitter and Facebook accounts exclusively created to reproduce Hache’s words.^1^

Steinberg already addressed the influence of media “for good, for bad, or for the ugly” (Macedo and Steinberg, 2007) and warned of the deleterious impact of commercial culture on children. She also explored these harmful effects on young girls with regard to gender and sexuality (Steinberg and Kincheloe, 1998). Along the same vein, our analysis of the film’s communicative acts uncovers the connection between supposed “love relationships” and the violence that has been identified in research on violence against women (Town and Adams, 2000; Borochowitz and Eisikovits, 2002). Nevertheless, these and other findings lead us to argue that 3MSC is not actually a “love story” but is instead a simple reproduction of the coercive dominant discourse, which links attractiveness to violent attitudes and behaviors (Puigvert et al., 2019). This model is based on a double standard (Gómez, 2014) that separates affection, stability, and friendship from excitement, arousal, and fun, and thus establishes a dichotomy between those to be in love and those to have passionate relationships that is evident and recurrent among adolescents and young women (Puigvert et al., 2019; Ruiz-Eugenio et al., 2020b). Hache’s character clearly belongs to the second group, as he is the archetype of Dominant Traditional Masculinity (DTM; Flecha et al., 2013). According to Gómez (2014) and contrary to popular belief and the image represented in the film, this type of man does not love or fall in love. As hunters, they are cold, calculating and fraudulent in their maneuvers to trap women.

The first objective of this article is to dismantle DTM strategies through the analysis of the communicative acts in 3MSC referred to and performed by Hache (presented in the section “Dismantling Hache’s Attractiveness in 3MSC”). In line with Subero et al. (2015) claim for the recognition of the multiple literacies that emerge from using different semiotic resources in different contexts of life and activity, research on hegemonic masculinity and its representation in mainstream media discourse has focused on the use of semiotic resources such as body image and language to reproduce heterosexual norms (Kivel and Johnson, 2009; Hiramoto, 2010) and the relation of this process to the “social semiotics of desire” (Cameron and Kulick, 2003). The semiotic notion of intertextuality (Kristeva, 1980) provided an explanatory basis for the mutual influence of text and context. Thus, we can argue that 3MSC depicts intertextual discursive practices that connect its main characters, Hache and Babi, with the hero and babe archetypes of heterosexual normativity- note that even their names evoke the archetypes- as described by Eckert and McConnell-Ginet (2003). These authors also explained the link between the so called heterosexual market (Thorne, 1993), its importance in the emerging adolescent social order and its influence on the way teenagers mold themselves as objects of desire to others (Eckert and McConnell-Ginet, 2003). Furthermore, Williams and Harper (2014) also noted that many research studies identified media as the origin of “gendered sexual scripts,” or the understandable conventions that organize sexual encounters (Simon and Gagnon, 1986), so that these “scripts” effectively dictate who will do what and when in a particular context. Recent contributions from Socioneuroscience show that the more intense the socialization linked to the dominant coercive discourse, the more emotional reactions of attraction to aggressive or violent men will occur (Puigvert et al., 2019).

In this respect, research on preventive socialization of gender violence has contributed abundant empirical evidence about the influence of the abovementioned coercive dominant discourse among adolescents (Puigvert et al., 2019). This has meant that “bad guys,” or those who reflect the DTM model, are chiefly perceived as appealing, while “good guys” are perceived as good friends, but not desirable. This finding is consistent with the great success of Hache and the double standard “created by DTM men as part of their domination in our patriarchal societies” (Flecha et al., 2013, p. 100). However, New Alternative Masculinities (NAM) men are challenging this traditional and unsatisfactory model of affective-sexual relationships (Portell and Pulido, 2012).

Previous research points out that heterosexual young women tend to accept the symbolic violence in 3MSC (Cardona et al., 2019). On the other hand, various investigations framed in preventive socialization of gender violence have shown that interactions based on the language of desire (Puigvert, 2016; Rios-González et al., 2018) are capable of transforming preferences in adolescent women choices, thus freeing participants from the pressure exerted by the dominant coercive discourse (Flecha et al., 2011; Racionero-Plaza et al., 2020; Ruiz-Eugenio et al., 2020a).

In this line, the second objective of this article is to demonstrate that interventions with adolescents discussing movies like 3MSC, with the involvement of NAM and grounded in the language of desire (Puigvert, 2016), can transform the perception about the sexual-affective relationship in the movie. The communicative acts gathered for the discussion group with the girls showed that dialogs framed by scientific evidence on male and attraction models can counteract mainstream socialization on these issues. Thus, at the end of discussions like the one analyzed in this article’s section “Changing Language of Desire: The Adolescents’ Voices,” participants have more information to assess whether their own desires and choices are leading them toward stormy and even violent relationships or to fully satisfying and violence-free relationships.

## About 3MSC

The Spanish version of 3MSC was the highest-grossing Spanish movie in 2010. It presents a supposed love story between Hache and Babi, two teenagers who live in worlds that appear almost irreconcilable. She is a good high school student and the older daughter of an affluent family, while he is a tough and violent biker that wastes his time riding his bike at top speed and getting into trouble with his gang. They are attracted to each other and, despite many difficulties, begin to date. Everything seems perfect, until Hache blunders several times because of his aggressive and violent attitude. In the climax of the story, Hache even hits Babi’s face and this ends their relationship. Then, Hache is portrayed as a very sorry and shattered boy who is the victim of his past suffering and instincts.

To fully understand the analysis in the following sections, we provide a short presentation of the film’s main characters:

- Hache is the main male character, a biker who always wears a black leather jacket and rides without a helmet. He does not study or work and mixes with lower-class bikers who like to drink alcohol, hold illegal motorcycle races and party. They shape a dangerous environment in which Hache appears to be the toughest guy.
- Babi is the main female character, a pretty high-class student who wears a school uniform. She is virgin, unlike her best friend Catina, and she appears to be the perfect daughter: responsible, quiet, kind, sweet, and intelligent.
- Dani is Babi’s little sister. She is the crazy girl in her home and displays great admiration and desire for Hache. Her continuous phone conversations with her friends are an interesting narrative resource because in many cases Dani makes explicit her sister’s thoughts.
- Mara is Babi’s nemesis, a tough lower-class girl who used to have sex with Hache until he leaves her for Babi. She is hooked on Hache and accepts her secondary role, though she suffers a lot.
- Chico is the alternative to Hache presented by the movie. He and Babi are about to date at the beginning of the story. However, a series of circumstances lead him, Babi and Hache to a dark and deserted road on the outskirts of the city. Hache hits Chico and, when Babi tries to defend Chico by jumping on Hache’s back and shouting at him, Chico takes advantage of the situation and escapes without her. Hache’s sardonic comment (“And he bailed!”) to Babi after that sheds a very unflattering light on Chico, and presents him as a weak, selfish coward.

## Materials and Methods

The results presented in this article are based on two different studies: the analysis of the 2010 Spanish movie version of 3MSC and a communicative focus group (CFG) held to discuss the movie and adolescents’ attraction models. In both cases, the focus was on communicative acts because prior research demonstrated their strong influence on affective-sexual relationships and the construction of NAM (Searle and Soler, 2005).

### Analysis of the Movie 3MSC

The analysis of the movie revolves around the communicative acts that reflect excitement, desire, or attraction toward Hache and on Hache’s own communicative acts that reveal violence or domination. In short, it is about exploring how the 3MSC script is full of communicative acts that enhance DTM’s attractiveness. Similar to Wingard and Lovaas (2014), our detailed language-in-use analysis does not focus on isolated comments, but we take into account the sequential context of the segments in which communicative acts occurs. Therefore, we selected six segments (five developed in detail as examples and the other one alluded more concisely in section “Dismantling Hache’s Attractiveness in 3MSC”), which display communicative acts that clearly illustrate the following analysis categories:

- Snubs, taunts, and provocation to get the girl’s attention.
- Connection with delinquency and risk taking.
- The creation of doubts and insecurity.
- Winner, leader, and hero.
- Justification of violent behavior.
- Self-confidence.

Two more categories have been identified that do not enhance Hache’s attractiveness, but are useful to subvert it:

- Selfish interest and unequal starting points.
- Excitement only in hunting.

### Communicative Focus Group

The second study was based on the analysis conducted in the first study. We used this analysis to edit a 18 min video with some of the most illustrative scenes in this respect together with others that are important to understand the 3MSC story as a whole. With the aim to dismantle the strategies that present Hache as attractive, we wrote short and forceful messages trying to reveal and, if possible, ridicule the strategy at stake. These comments were added to the selected scenes, generally at the end, to bring the strategies and the arguments against them face to face.

This video was shown to a group of eight female adolescents (ages 15–16) who participated in the CFG. Since the research focused exclusively on heterosexual affective-sexual relationships and more specifically on the attraction of young girls toward DTM men, participants were only heterosexual girls that have explicitly express that they liked very much the movie, and particularly Hache, the male leading role representing the paradigm of DTM men.

They all were classmates in a secondary education center. The CFG took place in one of their school’s classrooms and in the presence of one of their male teachers. The girls asked for their parent’s permission to participate and attended the school in their free time to participate in this discussion about the boys they feel attracted to and why. Additionally, the teacher and the researcher both had previous experience in discussions and workshops with adolescents about attraction models and NAM, so they could create a climate of confidence in which the girls could talk clearly and honestly.

The CFG lasted an hour and a half and followed this outline: presentation of the masculinity models and preliminary questions, viewing of the 18 min edited video about 3MSC, discussion about Hache and the DTM boys, viewing of another 11 min video (about the film “The Lucky One” and specially edited to display the attractiveness of a NAM character named Logan), discussion and comparison of DTM and NAM characters. The use of these materials and their subsequent discussion is consistent with the role a researcher is supposed to play in CFG, that is, “to motivate the participants of the group to engage in the debate and to bring their arguments – their lifeworld” (Díez-Palomar et al., 2014). At the same time, the researcher is also charged with bringing scientific evidence on the topic into the discussion (Martí and Mertens, 2014).

This data collection technique was selected because of its suitability for this study. Traditional focus groups are stressed by Click et al. (2014) for understanding how audiences make sense of TV because discussion offers a better sense of participants’ perspectives. Moreover, the communicative methodology of research has proved to be very effective for the identification and analysis of some elements underlying violence against women and has had an important impact in transforming situations of inequality (Gómez et al., 2011). Accordingly, the CFG aims not only to help in the collective generation of scientific knowledge through egalitarian dialog between researchers and participants but also to transform the context through this process of reflection (Aubert et al., 2011; Ruiz-Eugenio et al., 2020a). In this case, the second objective is to transform adolescents’ perceptions of the affective-sexual relationship depicted in 3MSC and, by extension, to question the mainstream attraction model that favors DTM boys.

The analysis of the data collected in the CFG (see section “Changing Language of Desire: The Adolescents’ Voices”) principally involved two categories: “perspectives on Hache’s violence” and the “division between boys desired for one night and boys for long-lasting relationships.” According to the communicative methodology, the data analysis also took into account the exclusionary and transformative components of the communicative acts collected. The text does not formally separate these two dimensions, but the analysis is presented chronologically, to better suit proposal of detailed language-in-use analysis of Wingard and Lovaas (2014), which focuses on the sequential context of the segments in which communicative acts occur.

### Ethical Considerations

The research team ensured all significant ethical standards, including informing participants of the aim of the study and procedures, also in terms of confidentiality, coding CFG and anonymization of participant information. Ethical approval for the study was obtained by the research ethics committee of CREA Community of Research on Excellence for All.

## Results

### Dismantling Hache’s Attractiveness in 3MSC

In this section, we discuss our analysis of the communicative acts displayed in 3MSC that enhance the attractiveness of Hache and, by extension, of DTM men. The order in which, we present the different examples is the same order that they appear in the movie. Some refer to the speech acts of Hache himself and others are the acts of other characters, but in all cases the narrative language employed in the film uses many semiotic resources (music, sound effects, locations, the characters’ clothes, gestures and tone of voice or even the context in which they take place) to shape communicative acts full of the language of desire (Flecha and Puigvert, 2010). This element has proved to be essential to both the analysis of the origins of violence against women and to its prevention.

Example 1 describes the communicative acts when Hache and Babi first meet:

1. Hache has just left the court where he was condemned for brutal aggression against his mother’s lover. His voiceover expresses his bad feelings and his disdain toward the people surrounding him: the victim of his aggression, the judge, his father, and his brother. He starts his powerful and customized motorbike and rides at high speed. He does not use a helmet and wears his characteristic black leather jacket half-opened.
  When he is passing by some cars stopped by the traffic lights and is about to see Babi, his voiceover says:
  “And suddenly it happens: something triggers and in that moment you know that things are going to change. They have already changed.”
  Then, Hache sees Babi, who is in the backseat of her father’s car, with her head out of the window, stops his motorbike and screams at her aggressively:
  Hache: Ugly! (Whistles at her) Yes, you!
  Babi puts her head back into the car. She looks astonished. Then, Hache pulls his motorbike up next to Babi’s car, touches her hand and laughs at her repeating:
  Hache: Ugly, what?….
  Babi quickly separates her hand from Hache’s and looks angry and surprised at the same time. When they are moving away, Babi puts her head again out of the window and gives the finger to Hache (this gesture means fuck you). However, when she puts her head again into the car, Babi’s expression changes from anger to excitement and pleasure, and she even smiles a little at the blank look on his face at the end of the scene.

This scene reveals a common DTM tactic, which consists of attracting the girl’s attention through snubs, taunts, and provocation. Moreover, these forms of mistreatment are usually conducted in a way that makes the girl feel insecure. In the example, Babi probably always thought she was pretty, but suddenly a stranger appears and screams “ugly” at her in such an aggressive and intrusive way. This strategy is always conducted by boys who try to portray the self-image of being untouchable and bastard. Research relates these features to boys who usually are considered very attractive by adolescents (Puigvert et al., 2019) and is consistent with the purpose of this strategy: to get the girl by making her feel she is the chosen one. In fact, Babi’s reaction demonstrates that the tactic worked. Situations like the one described in this example illustrate how mainstream socialization influences the separation of the language of ethics and the language of desire (Aubert et al., 2011), a matter that lays at the foundation of the double standard. DTM’s behavior is not perceived as good (at first, Babi rejects him), but it provokes the arousal of desire.

In addition to the strategies of the DTM character, the situation described above also shapes the communicative acts that contribute to DTM’s attractiveness. The fact that the judge considers Hache guilty for assault plus his riding on a powerful motorcycle without a helmet at high speed connects him with delinquency and risk taking. These two elements attract the romantic interest of adolescents, according to Rebellon and Manasse (2004).

Attraction to the untouchable and aloof boy is also evident in another scene in which Mara (“the other girl”) is talking to a friend about Hache. She went to bed with him twice and she is looking forward to receiving a phone call from him. Her friend argues that having had sex with him a couple of times does not mean they are together. However, Mara tries to justify Hache’s inattention because “he never calls.”. At that very moment, Hache arrives riding his motorcycle and Mara shows her excitement by touching her friends’ arm nervously. Hache takes off his leather jacket, while he is walking and gives Mara the “great honor” of holding the jacket during the flexing exercises contest in which he becomes the winner in front of the whole gang. Mara’s facial expression after Hache’s victory, which shows great joy, pride and desire illustrates how DTM’s strategy of creating doubt (Mara never knows if Hache is going to meet her again or if he is dating other girls) can generate excitement and attraction in addition to anguish and suffering. Mainstream socialization in sexual-affective relations has a great impact on Mara’s election and attraction (Gómez, 2014) because doubt, anguish, and suffering do not lead her to break up with Hache, but actually create more excitement.

This scene identifies Hache as a winner, a leader that exercises influence over the others. Leadership is also attributed to attractive boys and this feature also connects with the hero character referenced frequently in research on media and hegemonic masculinity (Kivel and Johnson, 2009; Hiramoto, 2010). Nevertheless, it is important to make clear that, unlike many heroes represented in cultural texts throughout history, DTM men link leadership with dominance over the others, so that they generate fear and submission (Valls et al., 2008). 3MSC tries to identify Hache as a hero through diverse communicative acts, like those exposed in the following example 2:

1. Hache and his gang burst into a party in the high-class neighborhood, where Babi is one of the guests. When Hache sees her, he repeats the strategy of taunting and provoking to attract her attention. She finally reacts by throwing her milkshake into Hache’s face. Hache responds violently by catching Babi and placing her on his shoulders, while pushing Chico to the ground. Then, Hache dives into the swimming pool carrying Babi on his shoulders.
  When Chico is later driving Babi home in his car, they are attacked by Hache’s gang on their motorbikes. The chase ends with Chico running away from Hache, who was beating him, leaving Babi alone with Hache in the dark and on a dangerous road far from the city. Finally, after facing another cocky boy who addresses Babi cheekily, Hache drives Babi home. He is wearing only his leather jacket because he got wet in the swimming pool. Babi’s dress is also wet, making her sylphlike body curve more evident.
  When they arrive, they meet Babi’s parents and sister, Dani. She looks at him lewdly from top to bottom and greets him. Later, when they are alone:
  Dani: (very excited and nervous, showing admiration to Babi) What are you doing with him? Are you going out with him?
  Babi: (annoyed) I do not even know him….
  Dani: (with enthusiasm) His name is Hugo Olivera, but he is called Hache. They say something terrible happened to him and that he wants to forget his name. My friends and I say that he is Hache, the hero. He seems like he saved your life….

This example provides several different elements that are worthy of analysis. First, the characterization of the hero as tough and aggressive has proven to be appealing for many adolescents and the general audience (Hiramoto, 2010; Click et al., 2014). Second, heroes are sexually desired by women, so “being a hero and being strong will get you the ‘girl’ in the end” (Kivel and Johnson, 2009; McDonald, 2015). Third, communicative acts within the peer group, represented by Dani, exert a great influence on adolescents’ tastes and preferences because they are performed through the language of desire. The idea of the protector who could “save your life,” as referenced by Dani refers to the moment in which Hache faces another cocky boy supposedly to keep Babi safe from him. It is worth paying more attention to this fragment of the scene previously narrated, but exposed more in detail in the following example 3:

1. Chico has just run away from Hache and left Babi alone with him on a dark and deserted road outside of the city. Babi is completely wet and her wet dress makes her sylphlike body curve more evident. Desperate, she tries to stop a car to get a ride home. A big customized red car with a big spoiler stops and the driver, showing big tattoos in his arm leaning on the car window, addresses Babi cheekily:
  Driver: Do you want me to take you somewhere, babe?
  Hache: (behind Babi, sitting on his bike, with a strong bully’s attitude, in loud voice) And do you want me to wring your neck, twat? Go, go away!
  The driver looks afraid and he immediately leaves. Hache and Babi are alone again. Babi is scared and wants to go home.
  Hache: (addressing Babi) Go, hop on! I have already fought enough people tonight because of you.
  Then, he drives Babi home safe and sound.

When Hache threatens the other cocky boy, the threat of violence pretends to be legitimate here because it is for a noble cause (Kivel and Johnson, 2009), to protect Babi from dangerous boys. This situation hints at a great paradox: although all men that are violent against women respond to the DTM model represented by Hache, the analysis of examples 2 and 3 reveal that the movie portrays Hache like a protective hero. However, in reality Hache himself created the mess and extreme circumstances that led to the dangerous situation for Babi. Presenting Hache as the rescuer from the danger he created is a rough strategy to enhance his attractiveness.

Self-confidence is another characteristic that generates attractiveness in men (Flecha et al., 2013; Castro and Mara, 2014; Joanpere and Morlà, 2019), so in 3MSC it is linked to DTM. Example 4 portrays some of the communicative acts that enhance this feature in Hache.

1. Hache hits a man (Mr. Santamaría), an acquaintance of Babi’s family, in the presence of Babi. The man wanted to file a law suit against Hache and asked Babi’s family for his name. Under her mother’s pressure, Babi informed against Hache. With Hache’s criminal record, another sentence would land him in prison.
  After all these events, Hache and Babi meet again incidentally in the place where illegal motorcycle races are held. When Hache sees Babi, he taunts and provokes her again. She also responds aggressively:
  Babi: Let us see if you are so cocky when you get the denunciation, because this afternoon I reported that it was you who broke Mr. Santamaría’s nose! (Sound effects emphasizing the gravity of the situation).
  On hearing these words, a friend of Hache insults Babi and pretends to attack her. He knows that his friend can go to jail. However, Hache stops and calms him with a self-confident tone of voice and affectionate gestures. Then, he continues, addressing his friend, but in loud voice and walking toward Babi:
  Hache: The day I will be called to testify this good girl will say that I did not do anything (Very close to Babi and addressing her full of confidence). Do you know why?
  Babi: (very nervous and flooded) Why?
  Hache: (staring at Babi with a half-smile) Because that day you will be so crazy for me that you could do anything to save me.
  Babi can only stare at him, totally flooded.
  That same night, a series of circumstances force Babi and Hache to run away together from the police. During the escape, Babi loses her clothes and stays in underwear. Hache lends his leather jacket to Babi and drives her home safe on his bike again. The music and the way Babi hugs Hache and lays her head on his back with their eyes shut suggest that she is about to drown in her desires. At the same time, Hache’s facial expression also reveals relaxation and satisfaction.

This scene not only depicts some of DTM’s strategies to get the girl but also that the whole process of seduction is in response to his interest in avoiding prison. Herein lies the importance of analyzing communicative acts because they take into account speech acts and body language as well as the role of interactions and the context of the moment in which the communication is produced. If attention is not given to these elements, which clearly set the two protagonists on unequal starting ground, one could believe that, despite all, they really like each other. Actually, the music, the scenery and the character’s expressions along the last ride described in the example above lead the audience to think that mutual feelings of love are growing between them. Nevertheless, in accordance with mainstream socialization in the coercive dominant discourse of affective-sexual relationships, Babi latches onto Hache, but not the other way round. As hunters, DTM men do not feel excitement in being with their prey, but only in hunting them (Gómez, 2014). The communicative acts in the next example (5), which occur immediately after those described in the previous one, are very illustrative in this respect:

1. When they arrive at Babi’s door, Hache takes Babi from his arm and then slides his hand until hers, very serious and staring at her with desire in his eyes. She corresponds grasping sweetly one of Hache’s finger with her hands, also staring at him. Their gazes are full of desire and excitement. Hache grabs Babi by her waist and moves her closer to him. She shivers and emits a labored moan, while he slides his hand up on her side. Hache puts his mouth close to Babi’s ear and whispers:
  Hache: Are you going to inform against me?
  Babi: Yes (also whispering, with haunting gaze, and softly nodding. She is almost numb with excitement).
  Hache: (Whispering) Yes? Do you swear to me?
  She cannot respond. He moves her hair away from her cheek and begins to kiss her, getting close to her mouth. Their lips are almost grazing and then Babi, with her eyes shut, slightly opens her mouth, absolutely devoted to passion. At this moment Hache separates his face, taunting her.
  Hache: Oh, Babi, Babi, Babi! I am a pig, an animal, a beast, a violent guy, but you would let me to kiss you (Snaps his tongue and shakes his hand with the index finger raised as a sign of denial). You are an incoherent.
  Babi: (Raging) And you are a bastard! (Leaving).
  Hache: (Chasing and taunting her) But, what were you doing there, gaping like a little fish and begging to be snogged?
  Babi turns around and slaps Hache’s face.
  Hache: Uuuuh! (In louder voice) I want you to give me my leather jacket back! Come on!
  Babi removes the jacket and throws it to Hache aggressively. She is very angry.
  Hache: Little fish! Are not you going to give a goodnight kiss to me?
  Babi: (Shouting) Go fuck yourself! (She leaves and slams the door).
  Hache stands on the other side of the door, smiling. His expression denotes that he has already got the girl.

This key moment depicts the success of all the DTM strategies to get the girl. The evident contrast between the completely committed girl, who is looking forward to being kissed, and the aloof boy that takes advantage of the girl’s desires to humiliate her reveals the irreconcilable opposition between what really excites each one of them. Yet 3MSC subsequent communicative acts try to show a beautiful and tender love story that would be impossible to believe using this analysis. First, because a relationship based on taunting, provocation, mistreatment, and constant tension cannot suddenly turn into one based on confidence, commitment, and passion with the same person. Second, because one of the parties has a selfish motive in being with the other person (not being denounced; Simmel, 1906). Third and finally, because DTM men do not fall in love or love women (Gómez, 2014), so that all communicative acts, which show Hache as a committed lover or that make Babi feel three steps above heaven are a farce.

### Changing Language of Desire: The Adolescents’ Voices

Communicative acts performed in dialogic contexts like the CFG “develop interactions that strengthen attraction toward persons with egalitarian values and that question the attractiveness of those who exercise power and domination” (Aubert et al., 2011, p. 302), so that they contribute to changing adolescents’ language of desire. Along the same lines, the CFG conducted in our study consisted of viewing the videos and a follow up. Before viewing the videos, the CFG participants were asked if they were attracted to Hache. They answered with an emphatic and unanimous “yes,” and some saying “of course.” Later, after viewing just the video, and being asked if Hache seemed attractive to them along the different scenes displayed, no one said “yes.” In fact, many of them said “no,” although lacking conviction, and one said “not at all.” This first change reflects the questioning of their initial preferences, but not a profound transformation. Other communicative acts occurred in the framework of the CFG, which we analyze below. Special attention is given to the two issues that appeared in the debate and that promoted relevant discussion for our analysis: the diverse perspectives on Hache’s violence and the division between boys desired for one night and boys for long-lasting relationships.

#### Perspectives on Hache’s Violence

The girls were asked for the most characteristic feature of Hache after viewing the 3MSC’s scenes specially edited for the occasion. It is worth noting that this video emphasized Hache’s violent behavior and strategies to get Babi. “Aggressive,” “cocky,” “hot-headed,” and “slap-happy” were the first answers, but immediately after Girl 1 took the floor:

1. Girl 1: He also has a past. He is aggressive for one reason… (the video shows that Hache committed his first brutal assault as a response to the discovery of his mother cheating on his father with the downstairs neighbor. The neighbor was the victim of Hache’s aggression and denounced Hache).
  Girl 2: Yes.
  Researcher: (ironically) Of course. And as he has that reason, what should we do with him?
  Girl 1: (doubting) Ok, yes, he has the reason…, he does not deserve the podium, but… I do not know…, you feel sorry for him.
  Girl 3: But, you cannot let him hit you because he has a past (…). A person who loves you very much is supposed not to treat you badly, because if he appreciates you, he cannot hit you, you know? So, if I demonstrate that I love you and you love me, he is not treating me badly…. It is assumed….
  Researcher: (Ironically) But, if he has a past, he suffered a lot and he is aggressive and treats you badly because he… like him (referring to Hache), he has a trauma. Then, what do you do?
  Girl 4: But, if he really loves you, no matter what happened, it is not an excuse to treat you this way.
  Researcher (addressing all the girls): Do you agree?
  Girl 5: Yes.
  Girl 6: Yes.
  Girl 1 (doubtful and unwilling to change his mind): More or less….

Faced with violence, Girl 1 and Girl 2 focused on the reasons of the aggressor, while Girl 3 and Girl 4 focused on the mistreatment and its incompatibility with love, a dichotomy already identified in research (Borochowitz and Eisikovits, 2002; Enander, 2011). Later in the discussion, when Girl 2 expressed her doubts about how to forecast whether a boy is going to hit you in the future, Girl 4 and Girl 3’s previous emphasis on the boy’s behavior made it easier for the researcher to contribute the scientific knowledge on masculinity models. Thus, paying attention to how a boy treats others, kindly or not, aggressively or not, was presented as a good predictor of how he would treat you. At the end of the conversation, these arguments appeared again when talking about how to choose with whom to have a relationship. First, some of the girls said they look at whether the boy is good-looking and his style of dressing, but immediately after others added elements like his way of looking, attitude, behavior, or the way he treats others and the way he treats you. This was interesting because the girls included in their own discourse the knowledge derived from scientific evidence and they used these arguments for appraising a boy’s attractiveness.

#### Division Between Boys Desired for One Night and Boys for Long-Lasting Relationships

Most girls made a clear distinction, first, between funny (and attractive) and boring (non-attractive) boys and, second, between boys for one night and boys for stable relationships. The second separation was surprising because it was made after viewing the videos, which clearly identified the DTM (Hache) and NAM (Logan) boys, and having unanimously chosen Logan as their best option. However, when they were asked who would be their election for a one-night relationship, they chiefly opted for Hache. They thought that sex would be better with boys like Hache. When the researcher asked why, they answered:

1. Girl 3: Because he is rougher (…). You can tell he is not shy.
  Girl 2: You can tell that he did (sex) many times.
  Girl 7: As if he also has experience….
  Girl 5: Because he goes hard (laughs).

These arguments were based on myths and suppositions derived from the double standard: tough guys are more determined, sexual experts, and tough sex provides more sexual pleasure. Girl 1 even said that Logan (the NAM boy displayed in the other video) would be too dull for her. In her eyes, he was not tough enough.

Wanting to emphasize the language of desire, the researcher provided arguments to dismantle the double standard, which was deeply assumed by the participants. On one hand, he argued that boys who are committed and passionate with a girl, like NAM, logically provide more sexual satisfaction than selfish boys who mistreat girls. To reinforce this position, a sex scene was shown (not from 3MSC, but from other movie and suitable for the girls’ ages) in which the protagonist was a NAM and that clearly showed that the girl was having a very funny, passionate, and sexually satisfying time.

On the other hand, the researcher referred to his experience in boy’s changing rooms as a former football player and revealed the comments that DTM boys make about the girls they get (“whore,” “bitch”…). These contributions were consistent with the findings from the study of the phenomenon of the mirage of upward mobility (Puigvert, 2016), which is the mistaken perception of linking the fact of having a romantic relationship with people responding to DTM to raising their status and attractiveness, when in fact the contrary occurs, and both status and attractiveness decrease (Oliver, 2010-2012). In accordance with this scientific and personal evidence, he asked the girls to think about how Hache (as archetype of DTM) and Logan (NAM) would talk about them after being with them one night. They clearly and unanimously recognized that Hache would speak ill about them, while Logan would speak well. They also recognized some cases of the mirage of the upward mobility in their context. Later, at the end of the discussion, the researcher asked again who they would choose for one night: Hache or Logan. This time at least half of the girls preferred Logan.

1. Researcher (addressing Girl 3, one of the participants that first would choose Hache and later Logan for a one-night relation): You told me that you are now opting for Logan….
  Girl 3: Oh…. Because… I do not know… it is true so that… it is not necessary to be with a bad boy and all that to have a funny night, because… I mean… the good guy can make you to have fun equally (…). It is better to be with a person who treats you well than other that treats you badly and so that later Hache would say: “I fucked her, she is a bitch.” The other guy would not do that. I did not think about that. And that is true.
  Researcher: What made you to change your mind?
  Girl 3: So that then they speak ill of you.

Although Girl 3’s language of desire did not change along the CFG, the use of the language of desire has proven to be useful for changing her perception and choices because she did not want to appear less attractive. This last example emphasizes the usefulness of research evidence for preventive socialization of gender violence and communicative acts for questioning the perception of girls according to mainstream socialization and for protecting them from DTM boys.

## Conclusion

Our analysis of 3MSC’s communicative acts leads to the conclusion that they serve to promote the coercive dominant discourse, which links attractiveness with DTM. The film demonstrates that cultural texts can be instruments for normalizing violence and for making violent men appear attractive. Thus, language is used to reference social norms and point to the unsatisfactory traditional model of affective-sexual relationships, the double standard and the discriminatory patriarchal order that shelters violence against women. The dissemination of violent stories portrayed as love stories feeds the dominant coercive discourse, reinforces the double standard, and increases the risk of gender violence.

Considering the success that movies like 3MSC have among heterosexual teenage women and their impact in terms of attraction to violence, it becomes necessary to dismantle the false romantic appearance of movies that have nothing to do with love and to unveil the strategies used by DTMs to perpetuate relationships that are not based on love but on domination. The study of communicative acts, which takes into account verbal and non-verbal language and the interaction and context of communication, permits a deeper understanding of affective-sexual relationships. Accordingly, the analysis provided differences between strategies to “get girls” and interactions guided by love and affection.

Moreover, sharing this scientific evidence in a CFG with teenage girls and stressing the language of desire, proved to be effective for transforming adolescents’ perceptions of the “love story” displayed in the movie. Personal choices and perceptions with regard to affective-sexual relationships were also transformed in the CFG in some cases. However, we cannot firmly assert that changes in the language of desire (that is, a profound shift in the adolescents’ attraction from DTM to NAM boys) have occurred. Actually, some adolescents participating in the discussion expressed some resistance that revealed the need for conducting further research on communicative acts and preventive socialization of gender violence because more elements are demanded to counteract the forces of the mainstream, traditional model of sexual-affective relationships.

## Practical Implications

The results presented in this article suggest that it is necessary to continue analyzing movies – especially those with significant impact on teenage audiences- that mask relationships of domination and present them as loving relationships, mainly focusing on the identification of the domination strategies used by DTMs. Besides, it is advisable to set up dialog processes based on the language of desire with the involvement of NAMs to counteract the negative impact that the dominant coercive discourse exerts on adolescent women in terms of attraction to violence, using the critical analysis of this type of movies as a starting point.

## Limitations

Field work data presented in this research relies upon the focus group conducted with eight adolescent heterosexual girls who claimed feeling attracted to Hache. To overcome this limitation and enrich the data, future research should explore the opinions of other adolescent profiles, including boys who identify with DTMs or NAMs, different sexual orientations, and diverse perceptions about the characters in the film.

## Data Availability Statement

The datasets presented in this article are not readily available because of personal information. Requests to access the datasets should be directed to Esther Oliver-Pérez (estheroliver@ub.edu).

## Ethics Statement

The research team ensured all significant ethical standards, including informing participants of the aim of the study and procedures, also in terms of confidentiality, coding CFG and anonymization of participant information. Ethical approval for the study was obtained by the research ethics committee of CREA Community of Research on Excellence for All. Written informed consent to participate in this study was provided by the participants.

## Author Contributions

RR-M and EO-P conceived the original idea of the article. MC-S conducted the research and data collection process and wrote the first draft of the manuscript. LB-G revised and edited the final version of the manuscript. EO-P supervised the final version. All authors contributed to the article and approved the submitted version.

### Conflict of Interest

The authors declare that the research was conducted in the absence of any commercial or financial relationships that could be construed as a potential conflict of interest.

## Footnotes

^1^See examples at: http://frasesdelapelicula.com/tres-metros-sobre-el-cielo-3msc/ http://desmotivaciones.es/218992/3-metros-sobre-el-cielo https://twitter.com/FrasesDeHache https://www.facebook.com/FrasesDeHache
